# Epigenetic and Physiological Responses to Varying Root-Zone Temperatures in Greenhouse Rocket

**DOI:** 10.3390/genes13020364

**Published:** 2022-02-17

**Authors:** Aphrodite Tsaballa, Ilektra Sperdouli, Evangelia V. Avramidou, Ioannis Ganopoulos, Athanasios Koukounaras, Georgios K. Ntinas

**Affiliations:** 1 Institute of Plant Breeding and Genetic Resources, Hellenic Agricultural Organization (ELGO-Dimitra), Thermi, 57001 Thessaloniki, Greece; ilektras@bio.auth.gr (I.S.); giannis.ganopoulos@gmail.com (I.G.); gntinas@ipgrb.gr (G.K.N.); 2 Institute of Mediterranean Forest Ecosystems, Hellenic Agricultural Organization (ELGO-Dimitra), Terma Alkmanos, Ilisia, 11528 Athens, Greece; aevaggelia@yahoo.com; 3 Department of Horticulture, School of Agriculture, Aristotle University of Thessaloniki, 54124 Thessaloniki, Greece; thankou@agro.auth.gr

**Keywords:** hydroponic floating systems, *Eruca sativa*, MSAP analysis, photochemical efficiency, root-zone temperature sustainable production

## Abstract

Greenhouse production of baby leaf vegetables grown in hydroponic floating trays has become extremely popular in recent years. Rocket (*Eruca sativa* Mill.) can grow in temperatures varying between 10 and 20 °C; nevertheless, a root-zone temperature (RZT) range of 18–23 °C is considered optimal for high productivity, photosynthesis, and production of metabolites. Maintaining such temperatures in winter raises production costs and prevents sustainability. In this study, we tested the impact of lower RZT on plants’ status and recorded their responses while providing energy for heating using photovoltaic solar panels. We used three hydroponic tanks for cultivation; a non-heated (control) tank (12 °C) and two heated tanks; a solar panel-powered one (16 °C) and a public grid-powered one (22 °C). Methylation-sensitive amplified polymorphisms (MSAP) analysis of global methylation profiles and chlorophyll fluorescence analysis were employed to assess methylation and physiology levels of rocket leaves. We found that there is demethylation at 16 °C RZT in comparison to 22 °C RZT. Reduction of temperature at 12 °C did not reduce methylation levels further but rather increased them. Furthermore, at 16 °C, the effective quantum yield of photosystem II (PSII) photochemistry (ΦPSII) was significantly higher, with a higher PSII electron transport rate (ETR) and a significantly decreased non-regulated energy loss (ΦΝO), suggesting a better light energy use by rocket plants with higher photosynthetic performance. ΦPSII was significantly negatively correlated with DNA methylation levels. Our results show that at 16 °C RZT, where plants grow efficiently without being affected by the cold, DNA methylation and photosynthesis apparatus systems are altered. These findings corroborate previous results where hydroponic production of rocket at RZT of 16 °C is accompanied by sufficient yield showing that rocket can effectively grow in suboptimal yet sustainable root-zone temperatures.

## 1. Introduction

Hydroponic vegetable production has become extremely popular worldwide due to resources use efficiency and quality of produce [1]. The efficient management of nutrient supply, temperature, and humidity at the roots of growing vegetables is a major advantage of this production technique. Moreover, in a floating hydroponic system, where plants develop on floating polystyrene trays inside tanks with a recirculating nutrient solution [2], water waste is minimal and nutrients do not leak, thus preventing environmental pollution [3]. Baby leaf vegetables such as rocket (*E. sativa* Mill.) are particularly suitable for cultivation in floating disks. Cultivation of baby leaf vegetables in hydroponic floating systems is simple and cheap. Furthermore, it provides plants with uniformity in their development, growth, and harvesting times, plus better control over growing conditions. The temperature, composition, and pH of the nutrient solution are important factors that influence the yield and quality of the product [4]. Low temperatures in the growing medium may not only impair roots’ capacity to acquire nutrients but also have a significant impact on plants’ physiology, growth, and quality [4,5]. Low-temperature stress has been long linked to changes in the phenotype but also in the biochemical and molecular profile of plants.

Exposing warm cultivated plants to temporary (hours to days) low but above-freezing temperatures (cold or chilling stress), as well as the ultrastructural injuries caused by it, has been known to have one of the most significant environmental influences on plant physiological functioning, with the chloroplasts being the most affected organelle [6,7]. Previous observations have demonstrated that cold/chilling stress causes damage to the chloroplast ultrastructure, which results in chloroplast swelling and unstacking of the chloroplast grana [7]. Chilling stress reduces CO_2_ assimilation and stomatal conductance, resulting in impairment of photosynthesis and electron transport through the thylakoid membrane, leading to cellular damage [8,9]. Ideal photosynthetic functioning involves a balance between energy input and energy consumption. When plants are exposed to low temperatures, the balance between “source” and “sink” processes may be disrupted, eventually causing photoinhibition [10]. The surplus energy, which is not safely dissipated, can cause the overproduction of reactive oxygen species (ROS) such as superoxide anion, hydroxyl radical, or hydrogen peroxide, resulting in serious oxidative damage [8].

As plant production is forced by photosynthesis, the outcome of plant growth and development could be assessed by estimating photosynthetic performance [11]. During photosynthesis, light energy is converted into chemical energy by photosystem I (PSI) and photosystem II (PSII), which function in coordination [11,12]. Chlorophyll fluorescence analysis has been widely used as a very sensitive marker of photosynthetic efficiency. Data gained can be used to obtain knowledge about the condition of the photosynthetic apparatus and the consequences of environmental stress on plants [11,13].

In general, abiotic stress conditions, such as cold and drought, drive responses by plants that change their epigenetic patterns either at the gene level or vastly across genomes [14]. The distribution of epigenetic marks such as cytosine methylation and histone modifications throughout the genome, which in turn direct gene expression and keep genome integrity, is called epigenome [15]. Epigenetic modifications involve changes in DNA methylation, histone modifications, chromatin remodeling, and small RNA changes that cause heritable alterations in gene transcription [16]. These changes are maintained through mitosis and/or meiosis and are generally stably maintained in the cells even without the presence of the stimulus. DNA methylation in plants occurs in cytosines that are in CG, CHG, and CHH (H stands for A, T, or C) sequence contexts and is catalyzed by a specific family of enzymes, DNA methyltransferases. DNA demethylation occurs when there is a failure in the maintenance of DNA methylation after replication or when active enzymes remove 5-mC from the DNA [17]. There have been numerous reports where, under drought, salt, fluctuation of temperatures, and mineral stresses, plants respond by changing their epigenomes [18,19,20,21,22,23]. Plants growing in stressful environments may have modified their epigenomes in a way that allows them to adapt to and tolerate difficult conditions [21,24,25]. In *Brassica rapa* of the Brassicaceae family, methylation and demethylation were recorded while plants of a cold-acclimated cultivar were exposed to 4 °C for two weeks [24]. Similarly, in *Arabidopsis thaliana*, it was found that plants exposed to stresses, including cold stress (plants under 4 °C for 12 h during the night for one week), produced progenies that showed higher DNA methylation, providing evidence for an epigenetic mechanism of stress adaptation [26].

The methylation-sensitive amplified polymorphism (MSAP) method is an effective technique to assess the methylation status of a plant genome. MSAP derives from the standard amplified-fragment-length polymorphism (AFLP) technique. One rare cutter restriction enzyme *EcoRI* and two frequent cutters, methylation-sensitive restriction enzymes *HpaII* and *MspI*, are utilized. *HpaII* and *MSPI* are a pair of isoschizomers; i.e., they recognize the same target sequence 5′-CCGG-3′, but their sensitivity to methylation at the inner or outer cytosine is different. MSAPs have been widely used in a variety of studies in order to study the different performance of tobacco in different environments and at different development phases [27] and the effects of drought stress on epigenetic changes in perennial ryegrass [28], alfalfa [29], and rice [19], and impact of salt stress on DNA methylation changes in two rice genotypes and successive recovery [30]. Moreover, studies using the MSAP method were employed in order to study heritable epigenetic changes in cotton [31], rice [32], and maize [33]. Although there are nowadays plenty of techniques and procedures to assess and profile DNA methylation, the MSAP technique remains one of the most popular to detect DNA methylation alterations in abiotic stresses, as revealed by numerous publications [34,35,36,37,38,39]. 

The quality of plants that grow in hydroponic systems is strongly affected by both air and nutrient solution temperatures [4]. Rocket plants can grow in air temperatures between 10 °C and 25 °C. In the root zone and in floating systems, however, an optimal temperature range of 18–23 °C should be maintained to stop the fungal diseases and protect the production of secondary metabolites [5,40]. Furthermore, a root-zone temperature (RZT) of 20 °C was found to be appropriate for yield, high photosynthetic rate, and high mineral concentrations in rocket, even though the concentration of plant metabolites was not as high [41]. We have recently found that the management of RZT conditions leads to higher yields, making root temperature a useful tool for the increase in productivity of rocket [42]. However, during winter, the preservation of optimal RZT is achieved with the use of fossil fuels, which are expensive and have a high environmental impact, in Mediterranean greenhouses. The scope of this study was to explore whether lower RZTs, which are easily maintained and less energy-demanding, especially when energy is provided by solar panels, can cause significant effects on plants’ homeostasis and more specifically on their epigenetic and physiology status. This snapshot of plants’ conditions will shed light on how plants respond to these non-optimal growth conditions. Furthermore, it will unravel the mechanisms that help rocket plants to adapt to this situation. 

## 2. Materials and Methods

### 2.1. Experimental Set-Up and Plant Material

**Figure 1:**
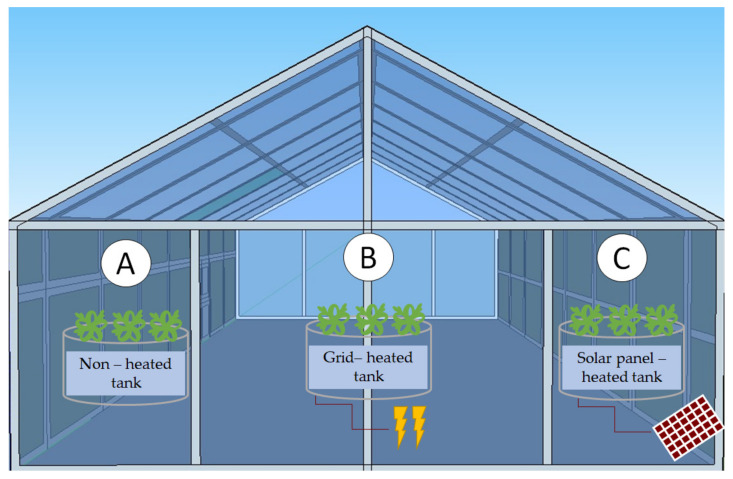
Experimental setup: rocket plants were grown inside hydroponic tanks with non-controlled (Tank A) and controlled (Tanks B and C) RZT. The difference between the two tanks with controlled RZT was the source of electrical power: solar panels vs. standard power grid.

Rocket plants were grown from commercially provided seeds (Geoponiki SA, Koropi Attiki, Greece) in hydroponic tanks inside a diffusion glass greenhouse at the Institute of Plant Breeding and Genetic Resources in Thessaloniki, Greece (40°32′17.4″ N, 22°59′58.2″ E), during a period between mid-January and early February 2019. Seeds were sown directly into Styrofoam rafts in peat moss substrate. Rafts with germinated plants at the stage of second true leaf were placed in the tanks to float after 14 days without transplanting. Natural ventilation was provided through roof openings without any other microclimate control during the experiment, which means that no air heating or cooling was provided inside the greenhouse during winter and early spring. Air temperature and humidity inside the greenhouse were recorded with a HOBO data logger (HOBO micro-station, 125 Onset Inc., Bourne, MA, USA). The three tanks used were made of galvanized steel and were covered with a low-density polyethylene (LDPE) sheet. Plants in each tank were supplied with 500 L of Hoagland nutrient solution produced from stock solutions and diluted with tap water. pH and EC values of the nutrient solution were 6.5 and 2.62 μS cm^−1^, respectively. The three hydroponic tanks were Tank A, where the RZT was not controlled; Tank B, where RZT was heated via the Greek power electricity grid; and Tank C, where the RZT control system was heated with electricity by a solar panel (photovoltaic—PV) system (Figure 1). The temperature inside the tanks was measured by PT−100 type sensors (Resistance thermometer Pt100, Uteco SA, Athens, Greece). More details can be found in [42]. An RZT of 22 °C is considered root-zone heating. The temperature in the tank powered by solar panels was around 16 °C, while the temperature in the control tank, which was unheated, did not exceed 12 °C. In the last two cases, the average temperature recorded at the plants’ root zone was lower than the optimum range (20–25 °C). Cold stress can be divided into chilling stress, when plants are grown around 0 to 15 °C, and freezing stress, when plants are grown in more severe conditions [43]. In our experiments, temperatures of 12 °C and 16 °C for the control and the solar panel-heated tank, respectively, were considered as root-zone chilling stress.

Leaves were collected from fully grown plants (just before harvest) in the non-heated tank (control), the tank that was heated by solar panels, with an average temperature of around 16 °C, and the tank heated by the grid, with an average temperature of 22 °C. Harvested leaves were blended, and soluble solids content (SSC) were measured by an Atago PR−1 handheld refractometer (Atago Co., Ltd., Tokyo, Japan), as a quality indicator. 

Leaves for DNA extraction were harvested in the morning at the developmental stage of four to five fully expanded leaves and when plants were of approx. 10 cm height. Sampling was performed as follows: each batch of leaves, collected from at least three different individual plants of one tray, selected at random, consisted of one replication; three replications per tank were used to prepare three separate DNA extractions (for an optical representation of sampling, see Supplementary Figure S1). DNA was extracted using an extraction buffer based on CTAB with the addition of polyvinyl pyrrolidone (PVP). Chloroform:isoamyl alcohol and isopropanol steps were performed afterward. The quantity and quality of the extracted DNA were estimated using a 1% agarose electrophoresis gel stained with ethidium bromide and Nanodrop spectrophotometer (Thermo Fisher Scientific, Waltham, MA, USA). Samples were then diluted to a 20 ng/μL working concentration to be used for the MSAP procedure. 

### 2.2. MSAP Procedure

From each sample, 500 ng of genomic DNA was digested with a 4U of *EcoRI* and 3U of *HpaII*, and 500 ng was treated with 4 U of *EcoRI* and 3U of *MspI*, for the MSAP procedure. The digestion was carried out at 37 °C for 3 h. The resulting digested DNA fragments and the *EcoRI* and *HpaII*/*MspI* adapters were ligated at 25 °C for three hours using 400 U/μL of T4 DNA ligase (New England Biolabs, Ipswich, MA, USA). Afterward, a heat shock treatment for 10 min at 65 °C to end the ligation reaction was performed. A primer pair based on the sequences of the *EcoRI* and *HpaII*/*MspI* adapters (Table 1) with one additional selective nucleotide at the 3′ end was used for the pre-selective PCR step. Pre-amplification PCR was performed in a total volume of 20 μL containing 1 × Kapa Taq Buffer, 0.4 u dNTPmix (10 mM), 2.5 mM MgCl_2_, 30 ng of each primer, 1U Taq DNA polymerase (Kapa Biosystems), and 5 μL of diluted fragments (from the digestion and ligation reaction). The cycling program was as follows: initially, a brief 30 s hold at 94 °C was implemented, followed by 23 cycles of 94 °C for 30 s, 56 °C for 30 s, and 72 °C for 1 min, followed by a final hold at 72 °C for 30 min. A 5 μL aliquot of the reaction was electrophoresed on agarose (1.25% *w*/*v* + ethidium bromide) to verify amplification; the remaining 15 μL was diluted 10-fold with TE. Selective amplifications were carried out in 10 μL total volumes containing 5 μL of diluted pre-selective template and 0.2 dNTP mix (10 mM), 2.5 mM MgCl_2_, 30 ng of *HpaII*/*MspI* primer, 30 ng of *EcoRI* primers (Table 1), and 1U of Taq DNA polymerase (Kapa Biosystems) per reaction. Selective amplification cycling was performed according to the following program: an initial cycle of 94 °C for 30 s, 65 °C for 30 s, 72 °C for 1 min, and then twelve cycles of 94 °C for 30 s, with an annealing temperature starting at 65 °C for 30 s, but decreasing by 0.70 °C for each cycle, 72 °C for 1 min, and finally, 22 cycles of 94 °C for 30 s, 56 °C for 30 s, 72 °C for 1 min, and a final hold at 72 °C for 30 min. Fragment separation and detection from selective amplification was performed in an ABI PRISM 3730xl DNA sequencer (Applied Biosystems, Waltham, MA, USA). 

### 2.3. Data Analysis

The presence or absence of fragments was indicated by the use of an AFLP Excel Macro that converted allele size data from GeneMapper4.0 (Applied Biosystems, Waltham, MA, USA) into binary form. The impact of potential size homoplasy was decreased by counting and analyzing only reproducible fragments ranging from 150 to 500 bases [44]. MSAP analyses were carried out as follows: evaluation of the banding patterns of *EcoRI*/*HpaII* and *EcoRI/MspI* reactions resulted in four conditions of a particular fragment; I: fragments present in both profiles (1/1), signifying an unmethylated state; II: fragments present only in *EcoRI/MspI* profiles (0/1), signifying hemi- or fully methylated CG-sites; III: fragments present only in *EcoRI/HpaII* profiles (1/0), signifying hemimethylated CHG-sites; and IV: absence of fragments in both profiles (0/0), representing an uninformative state produced either by different types of methylation or due to restriction site polymorphism [45]. Further analysis was performed in R software (https://www.r-project.org/, accessed on 10 January 2022) with MSAP_calc program and Mixed Scoring II method, which is a reliable program and is a method to retrieve different methylation changes [45]. Furthermore, GenAlEx 6 [46] was used for the analysis of each category of methylated (h and m) and non-methylated (u), alleles as well as the estimation of epigenetic Shannon Information Index (Iepi) and haploid epigenetic diversity (hepi) within and between the two treatments and the control. Significant differences between different treatments were calculated with the Kruskal–Wallis test. Moreover, Dunn’s post hoc tests with Bonferroni correction on each pair of groups for h, m, u alleles and total methylation (h + m alleles) for Iepi and hepi were carried out. 

### 2.4. Chlorophyll Fluorescence Measurements

Dark-adapted (15 min) rocket leaves of the same developmental stage and grown in non-heated control (12 °C) (Tank A), electricity-powered (22 °C) (Tank B), and solar panel-powered (16 °C) (Tank C) tanks were used for the measurement of chlorophyll fluorescence parameters. A chlorophyll fluorometer imaging-PAM M-Series (Heinz Walz GmbH, Effeltrich, Germany) was employed, and measurements were taken as described by Moustaka et al. [12]. Six leaves were measured from six different plants of each tank with an actinic light intensity of 436 μmol photons m^−2^ s^−1^. By using the Imaging Win software, we estimated the effective quantum yield of photochemistry in PSII (ΦPSII); the quantum yield of regulated non-photochemical energy loss in PSII (ΦNPQ); the quantum yield of non-regulated energy loss in PSII (ΦNO); the photochemical quenching (qp), which is the fraction of open PSII reaction centers; the non-photochemical quenching (NPQ); and the relative PSII electron transport rate (ETR). 

For statistical analysis, chlorophyll fluorescence measurements are introduced as the mean ± SD. Paired *t*-tests at a level of *p* < 0.05 were performed with the StatView software (computer package, Abacus Concepts, Inc., Berkley, CA, USA) as before [11,47,48]. 

## 3. Results and Discussion

In our experiment, rocket plants were grown in heated hydroponic tanks powered by electricity provided by the public grid and solar panels, while control rocket plants were grown in tanks that were not heated. 

To document the extent to which rocket plants experienced cold in their root zones when grown under suboptimal conditions inside heated and non-heated hydroponic tanks, and whether this affects their performance, we used different kinds of analysis. First, we used MSAP markers to investigate the variation in DNA methylation between samples. MSAP analysis of global genome methylation showed that significant differences were recorded in total methylation in rocket in response to chilling conditions. Since Shannon Diversity Index and genetic diversity are strongly correlated, and Shannon provides an alternative method of quantifying biological diversity across multiple scales, we further discuss results for Iepi. Additionally, Shannon Information Index (I) is a widely used measure of diversity in ecology since it computes allele differences across loci of various samples. More specifically, according to Dunn’s post hoc tests with Bonferroni correction for Shannon Information Index (Iepi) and haploid epigenetic diversity (hepi) on each pair of groups, we found that (Table 2):

(a) For h alleles, significant differences were found between plants grown in the non-heated control tank (12 °C) and the solar panel-heated tank (16 °C) and between the grid-heated tank (22 °C) and the solar panel-heated tank.
(b) For m alleles, significant differences were found between plants grown in the non-heated control tank and the solar panel-heated tank and between the grid-heated tank and the solar panel-heated tank.
(c) For total methylation (h + m), significant differences were found between plants grown in the non-heated control tank and the solar panel-heated tank and between plants grown in the grid-heated tank and the solar panel-heated tank.
(d) For u alleles, significant differences were found between the non-heated control tank and the grid-heated tank and between the control and the solar panel-heated tank. U alleles are the unmethylated alleles.

**Figure 2:**
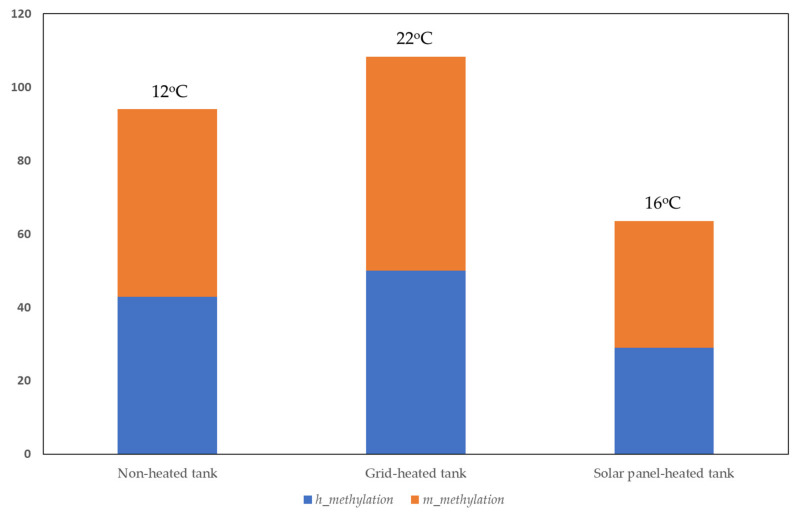
Total methylation patterns (h + m) based on MSAP analysis in control plants grown in non-heated tanks, in plants grown at an optimum of 22 °C in a grid-heated tank, and in plants grown in 16 °C in a solar panel-heated tank. Pooled leaves from at least three individual plants were analyzed for each sample/treatment. The Y-axis symbolizes total methylation, which derives from the sum of h + m epialleles, and is presented as a percentage.

Rocket plants grown in the solar panel-heated tank, where the RZT was 16 °C on average, had significantly lower total methylation than the control plants grown in colder conditions of 12 °C on average (Figure 2). On the contrary, plants grown in the grid-heated tank, where there was a constant of 22 °C in their root zone, had significantly higher total methylation levels compared to plants grown in the solar panel-heated tank. We recorded demethylation in plants grown at 16 °C in comparison to plants grown only under chilling conditions of 12 °C, but methylation levels were higher when plants were normally heated (Figure 2). The difference between methylation levels of the control and the grid-heated tank is not significant, so we could not correlate methylation levels with exposure to chilling conditions. However, given that 16 °C and 12 °C are sub-optimal chilling conditions that impose a stress on growing rocket roots, it is evident that there was a decrease in methylation in comparison to the optimal growing conditions.

DNA methylation changes in response to stresses play an important role in how plants respond and adapt to changes in their environment. DNA methylation alters gene transcription by creating heritable variation. Exposure to cold imposes a significant stress on plants, which “mobilize” their DNA methylation mechanisms to cope. By using MSAP markers in maize, it was found that DNA methylation is reduced early after 24 h exposure of seedlings to low temperatures, showing that plants’ response to the changing environment is fast in order to regulate gene expression at the seedling stage [20]. Variations in DNA methylation in rice cultivars exposed to cold stress that were associated with the developmental stage and the tissue or organ were also recorded [49]. In the same study, MSAP markers analysis revealed an increase in DNA methylation in cold-stressed plant roots in the cold-tolerant rice genotype. On the other hand, an increase in DNA methylation in the leaves and demethylation in the panicles of rice plants that belong to the non-tolerant genotype at the booting stage were recorded [49]. Maize and rice grow in warmer regions than Brassicas, but even species that are well-adapted to cold show distinct changes in DNA methylation after cold exposure, where predominantly DNA demethylation occurs [21]. This is in agreement with our results in which plants grown in sub-optimal chilling conditions have lower DNA methylation levels than plants grown in optimal conditions. Cold-acclimated genotypes seem to have different methylation profiles in comparison to non-acclimated ones, but the picture is not yet apparent; both DNA methylation and demethylation occur in various genomic locations in cold-acclimated *B. rapa* plants exposed to 4 °C for two weeks in comparison to nonacclimated controls. These plants have higher photosynthesis rates, indicating their acclimation and resulting in higher growth rates [24].

**Figure 3:**
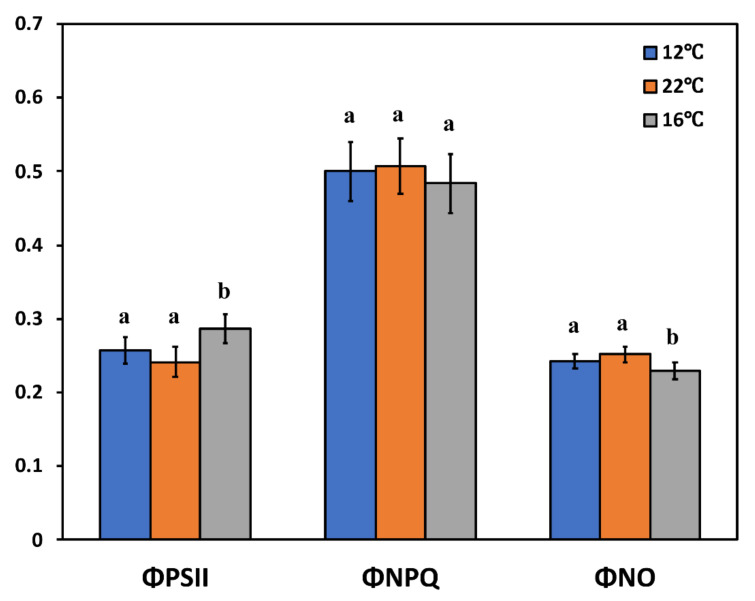
The quantum yields of photosystem II (PSII) photochemistry (ΦPSII), of regulated non-photochemical energy loss (ΦNPQ), and of non-regulated energy loss (ΦNO) of rocket plants grown in non-heated tanks (12 °C), in grid-heated tanks (22 °C), and in solar-panel-powered tanks (16 °C). Error bars on columns are standard deviations based on six leaves from different plants. Different letters represent a significantly different mean for the same parameter (*p* < 0.05).

The allocation of absorbed light energy in the rocket plants grown in tanks with different root-zone-controlled temperatures was assessed by measuring the fraction of the absorbed light energy used for photochemistry (ΦPSII), as well as the fraction that is lost by regulated heat dissipation (ΦNPQ) and non-regulated energy loss (ΦNO). In rocket plants grown in solar-powered tanks where RZT was 16 °C, ΦPSII was significantly increased compared to the plants grown in grid-powered (22 °C) and non-heated tanks (12 °C), indicating a higher fraction of absorbed light energy to be directed to photochemistry under 16 °C (Figure 3). Although there are no significant differences in the quantum yield of regulated heat dissipation in PSII (ΦNPQ) under all temperatures, ΦNO was significantly decreased in rocket plants grown in solar-heated tanks at 16 °C, showing a better use of the absorbed light energy (Figure 3). At RZTs of 22 °C and 12 °C, ΦPSII, ΦNPQ, and ΦNO did not show any significant differences.

**Figure 4:**
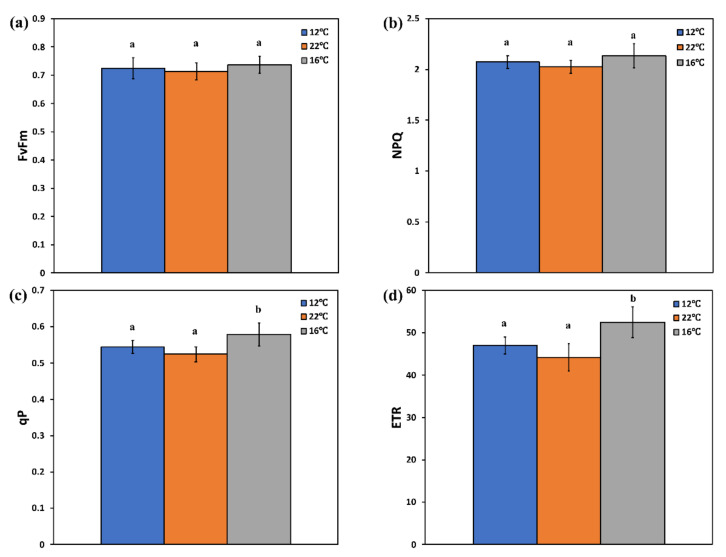
(**a**) The maximum efficiency of PSII photochemistry (Fv/Fm); (**b**) the non-photochemical quenching (NPQ) that reflects heat dissipation of excitation energy; (**c**) the photochemical quenching, which is the fraction of open PSII reaction centers (qp); and (**d**) the relative PSII electron transport rate (ETR) of rocket plants grown in non-heated tanks (12 °C), in grid-heated tanks (22 °C), and in solar panel-heated tanks (16 °C). Error bars on columns are standard deviations based on six leaves from different plants. Different letters represent a significantly different mean for the same parameter (*p* < 0.05).

The maximum efficiency of PSII photochemistry (Fv/Fm), as well as the non-photochemical quenching (NPQ), that reflects heat dissipation of excitation energy, did not differ among rocket plants grown either in solar-heated tanks (16 °C) or grid-heated (22 °C) and non-heated tanks (12 °C) (Figure 4a,b). Furthermore, in rocket plants grown at 16 °C, the fraction of open PSII reaction centers (qp) was significantly higher (Figure 4c), and PSII electron transport rate (ETR) followed the pattern of ΦPSΙI (Figure 4d). It has been shown that under sub-optimum growth temperatures, the Fv/Fm ratio and photosynthetic capacity decrease [50], but in our case, it seems that plants are not affected. In our experiment, the Fv/Fm ratio was not proven to be a sensitive indicator of the photosynthetic efficiency, as has been also pointed out in inoculated and non-inoculated *Salvia fruticosa* plants [47], and under paraquat toxicity [51], where ΦPSΙI was proposed as the most sensitive bioindicator. According to the model of PSII function proposed by Moustaka et al. [51], the efficiency of open reaction centers in the light (Fv’/Fm’) can be calculated by dividing ΦPSII by the fraction of open PSII reaction centers (qp). Thus, at 16 °C, the efficiency of open reaction centers was calculated to be 0.495, which is significantly higher than at 22 °C (0.461) and at 12 °C (0.472). Consequently, the increased ΦPSΙI at 16 °C was due to both an increased fraction of open reaction centers and an increased efficiency of those centers [52]. The significantly higher ETR in plants grown at 16 °C (Figure 4d) suggests an increased photosynthetic performance that provides growth advantages [53]. 

Linear correlations between total relative methylation levels, photosynthetic parameters, yield, and TSS show that there are statistically significant positive correlations between h methylation (hemimethylated CHG sites) and m methylation (fully methylated CG sites) (r = 0.999), as well as between h methylation and ΦNPQ (r = 1.000), ΦNO (r = 0.998) (Table 3). Similarly, m methylation CG is statistically positively correlated with ΦNPQ (r = 1.000). Statistically significant negative correlations exist between h methylation CHG and ΦPSII (r = −1.000), m methylation CG and ΦPSII (r = −0.998), ΦPSII and ΦNPQ, and ΦNO (r = −0.999 and r = −0.999). In general, methylation levels are positively correlated with yield, ΦNPQ, and ΦΝO and negatively correlated with TSS and ΦPSII. TSS is positively correlated with ΦPSII. Interestingly, the negative significant correlation between ΦPSII and DNA methylation levels shows that demethylation of DNA is possibly accompanied by a higher fraction of the absorbed light energy that is used for photochemistry, providing evidence that higher transcription of genes leads to higher photosynthetic efficiency. These results agree with previous results, where photosynthetic parameters such as net photosynthetic rate and stomatal conductance were positively correlated with total relative methylation level and hemimethylation in poplar (*Populus tormentosa*) [54]. We found a significant positive correlation between DNA methylation and non-regulated energy loss (ΦNO), suggesting a higher level of DNA methylation with increasing ΦNO. Hence, it is possible that alterations in DNA methylation influence the transcriptional component of the acclimation response, impairing the ability for the removal of excess excitation energy and thus resulting in increased singlet oxygen formation. ΦNO consists of chlorophyll fluorescence internal conversions and intersystem crossing, which results in ^1^O_2_ formation via the triplet state of chlorophyll (^3^chl*) [55,56]. 

Control of RZT using environmentally friendly techniques such as renewable energy from solar panels is a valuable tool for increasing yield in winter rocket floating-cultivation systems [42]. DNA methylation is an important epigenetic mechanism that plants utilize as a response to a variety of abiotic stresses. Heritable DNA variations create new phenotypic variations that can be valuable for plant breeding [57]. It is now proven that cold during the vernalization process drives changes in the epigenetic state of a specific gene in *Arabidopsis*, representing a form of epigenetic memory transmitted through mitosis of meristematic cells [58]. This is the first time that changes in DNA methylation have been recorded in rocket plants grown under chilling conditions in hydroponic tanks. Our molecular and physiology data show that plants change their DNA methylation and photosynthesis mechanisms, which possibly gives them the advantage of being able to cope with and adapt to colder temperatures and thus grow efficiently in tanks, even with lower RZT. The strong correlation between DNA methylation levels and photosynthetic performance parameters further supports this notion. MSAP analysis screens anonymous loci, and therefore, no conclusion can be drawn about the genomic regions or genes that are affected by methylation, nor on whether these regions are somehow related to photosynthetic performance or abiotic stress tolerance. However, given that plants use DNA demethylation mechanisms to initiate the transcription of genes in response to biotic and abiotic environmental cues [59], it is interesting to think that gene initiation at 16 °C takes place in rocket plants, and this might be linked to photosynthesis motivation. Nevertheless, the fact that yield is compromised at colder temperatures rules out the growth of rocket in non-heated tanks and shows that 16 °C might be a sufficient and adequate temperature of balance between the costly optimum temperatures (of 22 °C) and the temperatures (of 12 °C) that are harsh on productivity. The above results provide further evidence that rocket can be efficiently grown in solar-powered heated tanks even under suboptimal temperatures. Furthermore, they elucidate findings from previous work on rocket, where these growing conditions and the preservation of RZT around 16 °C has led to a significant 15.1% higher yield in comparison to non-heated tanks [42]. However, further experiments are needed to establish a causative relationship between methylation and yield.

## 4. Conclusions

In this study, it was found that greenhouse rocket plants with an RZT of 16 °C, as controlled by PV panels, demethylate their DNA compared to plants grown with an RZT of 22 °C. DNA was not demethylated further when the root zone was not heated. However, rocket plants made better use of absorbed light energy at 16 °C and increased photosynthetic performance due to their higher ETR and qp parameters, which means that the photosynthesis function provides them with growth advantages that they do not possess at lower temperatures. The registration of lower methylation levels and higher photosynthetic performance at 16 °C could be an indication of a possible adaptation of plants at this temperature. However, further research is needed to draw safer conclusions. 

The increased demand for healthy diets leads to year-round intensive vegetable production. Protected cultivation of vegetables in a controlled environment is required to deliver high-quality products. However, this leads to increased energy consumption and environmental burden. Modern agriculture needs to prioritize cost and sustainability. Growing rocket hydroponically at slightly lower temperatures by utilizing renewable energy sources can help towards achieving a lower-input agriculture with neutral environmental impact. Furthermore, it paves the way for stirring breeding through manipulating epigenetics and physiology mechanisms for better yield and climate adaptation. 

## Figures and Tables

**Table 1:** Adapters and Primers used for the MSAP analysis.

	5′ to 3′ Sequence
*EcoRI* adapter	CTCGTAGACTGCGTACC AATTGGTACGCAGTC
*HpaII*/*MspI* adapter	GACGATGAGTCTCGAT CGATCGAGACTCAT
Pre-selective *EcoRI* primer	GACTGCGTACCAATTC−A
Pre-selective *HpaII*/*MspI* primer	ATGAGTCTCGATCGG−T
Selective *EcoRI* primers	GACTGCGTACCAATTC+ATG GACTGCGTACCAATTC+ACT GACTGCGTACCAATTC+AAC GACTGCGTACCAATTC+AAG
Selective *HpaII*/*MspI* primer	ATGAGTCTCGATCGG+TCA ATGAGTCTCGATCGG+ACT ATGAGTCTCGATCGG+AAT

**Table 2:** Mean epigenetic Shannon Information Index (Iepi), epigenetic diversity (hepi) and standard error (SE) for the three tanks. A Kruskal–Wallis test provided very strong evidence for a difference (*p* < 0.001) between the mean ranks of at least one pair of groups for all comparisons for h, m, u, and total alleles. Dunn’s pairwise tests were carried out for these pairs of groups. Significant differences are provided based on Dunn’s post hoc tests with Bonferroni correction among treatments for each treatment and are indicated with the same letters. h alleles = hemimethylated, u alleles = unmethylated alleles, m alleles = methylated alleles.

		h Alleles		u Alleles		m Alleles		Total Methylation (h + m)	
		Iepi	Hepi	Iepi	Hepi	Iepi	Hepi	Iepi	Hepi
Tank A *	Mean	0.221 ^a^	0.143 ^a^	0.188 ^c,d^	0.125 ^c,d^	0.282 ^e^	0.188 ^e^	0.253 ^g^	0.167 ^g^
	SE	0.017	0.011	0.016	0.011	0.018	0.012	0.012	0.008
Tank B	Mean	0.254 ^b^	0.164 ^b^	0.252 ^c^	0.165 ^c^	0.321 ^f^	0.214 ^f^	0.289 ^h^	0.190 ^h^
	SE	0.017	0.011	0.017	0.011	0.017	0.012	0.012	0.008
Tank C	Mean	0.145 ^a,b^	0.093 ^a,b^	0.277 ^d^	0.186 ^d^	0.175 ^e,f^	0.113 ^e,f^	0.161 ^g,h^	0.104 ^g,h^
	SE	0.015	0.010	0.018	0.012	0.015	0.010	0.011	0.007

* Tank A = Non-heated control tank, Tank B = Electricity-heated tank, Tank C = Solar panel-heated tank.

**Table 3:** Linear correlations between total methylation levels (hemimethylated CHG + fully methylated CG sites) (based on MSAP analysis), photosynthetic parameters (ΦPSII, ΦNPQ and ΦNO), yield, measured in kg/m^2^ (Karnoutsos et al. [42]), and total soluble solids (expressed as %brix). Significant differences were examined using a *t*-test (* *p* < 0.05).

	h Methylation	m Methylation	Yield	TSS	ΦPSII	ΦNPQ
h methylation						
m methylation	0.999 *					
Yield	0.314	0.273				
TSS	−0.761	−0.732	−0.855			
ΦPSII	−1.000 *	−0.998 *	−0.337	0.776		
ΦNPQ	1.000 *	1.000 *	0.293	−0.746	−0.999 *	
ΦNO	0.998 *	0.995	0.368	−0.797	−0.999 *	0.997

## Data Availability

Not applicable.

## Supplementary Materials

The following supporting information can be downloaded at: https://www.mdpi.com/article/10.3390/genes13020364/s1, Figure S1: optical representation of sampling.
