# Supplementary figures and images for: Epigenetic and Physiological Responses to Varying Root-Zone Temperatures in Greenhouse Rocket
Source: Genes (Basel). 2022 Feb 17;13(2):364. doi: 10.3390/genes13020364 (PMC8871717; doi:10.3390/genes13020364)

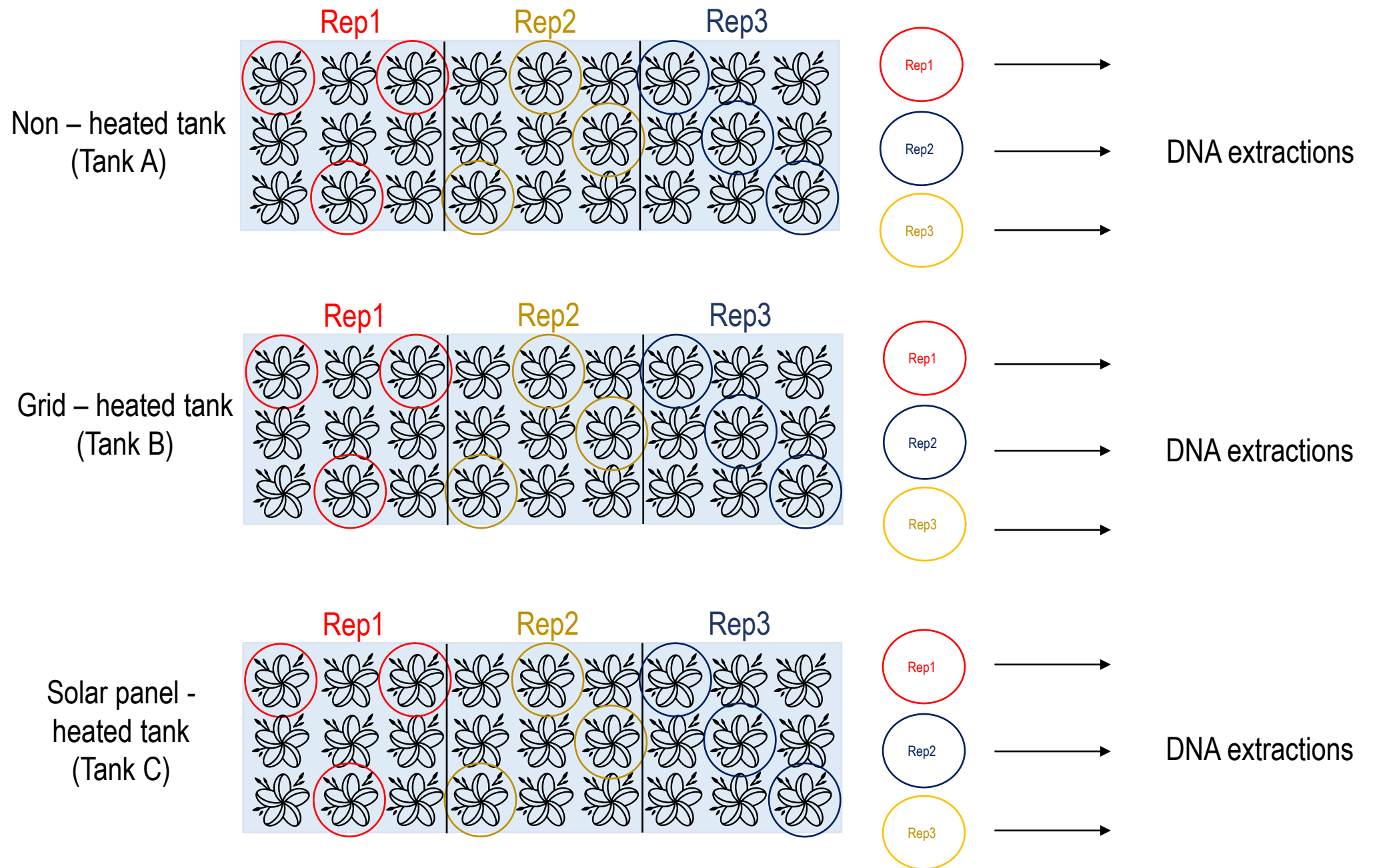

**Figure S1.** Optical representation of sampling.

Supplement: Supplementary file 1 [file genes-13-00364-s001.zip › genes-1572745-SI.pdf]
